# Sensing whales, storms, ships and earthquakes using an Arctic fibre optic cable

**DOI:** 10.1038/s41598-022-23606-x

**Published:** 2022-11-10

**Authors:** Martin Landrø, Léa Bouffaut, Hannah Joy Kriesell, John Robert Potter, Robin André Rørstadbotnen, Kittinat Taweesintananon, Ståle Emil Johansen, Jan Kristoffer Brenne, Aksel Haukanes, Olaf Schjelderup, Frode Storvik

**Affiliations:** 1 Acoustics Group, Department of Electronic Systems, Norwegian University of Science and Technology (NTNU), 7491 Trondheim, Norway; 2 Centre for Geophysical Forecasting, Norwegian University of Science and Technology (NTNU), 7491 Trondheim, Norway; 3 K. Lisa Yang Center for Conservation Bioacoustics, Cornell Lab of Ornithology, Cornell University, Ithaca, NY 14850 USA; 4 PTT Exploration and Production Public Company Limited, Bangkok, 10900 Thailand; 5 Department of Geoscience and Petroleum, Norwegian University of Science and Technology (NTNU), 7031 Trondheim, Norway; 6 Alcatel Submarine Networks Norway AS, 7075 Tiller, Norway; 7 Uninett AS (merged into Sikt in January 2022), 7030 Trondheim, Norway

**Keywords:** Marine mammals, Geophysics, Physical oceanography

## Abstract

Our oceans are critical to the health of our planet and its inhabitants. Increasing pressures on our marine environment are triggering an urgent need for continuous and comprehensive monitoring of the oceans and stressors, including anthropogenic activity. Current ocean observational systems are expensive and have limited temporal and spatial coverage. However, there exists a dense network of fibre-optic (FO) telecommunication cables, covering both deep ocean and coastal areas around the globe. FO cables have an untapped potential for advanced acoustic sensing that, with recent technological break-throughs, can now fill many gaps in quantitative ocean monitoring. Here we show for the first time that an advanced distributed acoustic sensing (DAS) interrogator can be used to capture a broad range of acoustic phenomena with unprecedented signal-to-noise ratios and distances. We have detected, tracked, and identified whales, storms, ships, and earthquakes. We live-streamed 250 TB of DAS data from Svalbard to mid-Norway via Uninett’s research network over 44 days; a first step towards real-time processing and distribution. Our findings demonstrate the potential for a global Earth-Ocean-Atmosphere-Space DAS monitoring network with multiple applications, e.g. marine mammal forecasting combined with ship tracking, to avoid ship strikes. By including automated processing and fusion with other remote-sensing data (automated identification systems, satellites, etc.), a low-cost ubiquitous real-time monitoring network with vastly improved coverage and resolution is within reach. We anticipate that this is a game-changer in establishing a global observatory for Ocean-Earth sciences that will mitigate current spatial sampling gaps. Our pilot test confirms the viability of this ‘cloud-observatory’ concept.

## Introduction

**Figure 1:**
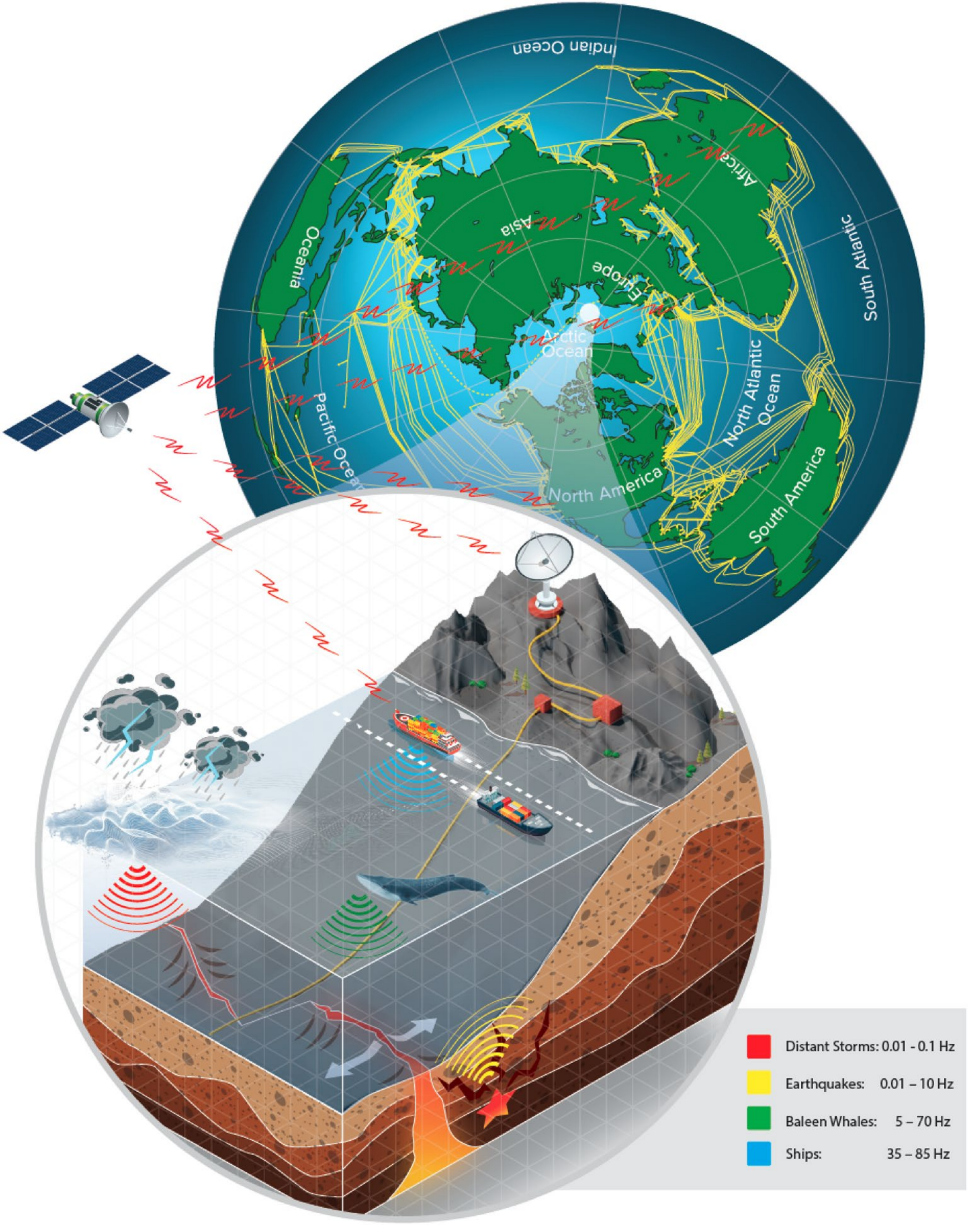
Earth-Ocean-Atmosphere-Space observatory concept. North polar stereographic projection of the world in the upper-right-hand globe, showing the extensive network of existing Fibre-Optic (FO) cables (yellow lines). The lower-left zoomed inset illustrates the key features of the observatory and its capabilities, including the DAS detection, tracking and identification of whales, ships, storms and earthquakes, processed in real-time, fused with other sensing sources such as satellite-derived Automated Identification System data from ships and relayed to the cloud. The frequencies associated with the various sources are those used in this paper whereas actual phenomena span a much wider frequency range, and indeed DAS can be used up to at least 5 kHz.

As awareness of the importance and complexity of the ocean environment becomes increasingly clear, the need for improved sensing, with better spatial resolution and increased temporal coverage, is now pressing^1^. In this article, we demonstrate that standard Fibre-Optic (FO) telecommunication cables, of which there are already 1.2 million km that crisscross the world’s oceans (Fig. 1, enough to wrap around the Earth 30 times), provide a means to continuously measure low-frequency acoustic events over large distances via Distributed Acoustic Sensing (DAS). DAS measures strain modulations in FO cables based on the inherent backscatter in optical fibres but has so far not been used to collect field data with such quality and range along the FO cable as we report here. With this increased range and signal quality, we show that such cables offer a unique potential for a cloud-based, global Earth-Ocean-Atmosphere-Space observatory that combines in-situ high spatial resolution DAS data from around the world with satellite and other remote sensing systems, a concept illustrated in Fig. 1.

Over the last decade, DAS has received growing attention mainly because of its potential to instrument areas with little or no current coverage, e.g., oceanic tectonic regions. The first study by Molenaar et al.^2^ used DAS in a geophysical application to monitor hydraulic fracturing in real time. Half a decade later, Lindsey et al.^3^ reported the first earthquake signals observed on horizontal FO cables, followed by Jousset et al.^4^ who examined structural features near Reykjanes peninsula in Iceland using local earthquakes and ambient noise, Ajo-Franklin et al.^5^ who analysed earthquakes on regional to teleseismic scale on a dark FO cable in California, and Williams et al.^6^ who studied a teleseismic earthquake in Belgium as well as low-frequency ocean signals. In addition to earthquakes, DAS in ocean-bottom telecommunication cables can detect ocean surface gravity waves and microseisms^6–9^. However, no attempt has been made to study the origins of ocean surface gravity waves from DAS data. Data with long recording duration are necessary for studying the dynamics of ocean surface gravity waves originating from distant storms. For example, Zhan et al.^10^ showed several dispersive signals associated with ocean swells from distant storms in a spectrogram computed over 11 days of FO sensing data. The increased spatial coverage and bandwidth offered by the recent generation of DAS instrumentation enabled studies of water-borne acoustic sources^11^. Rivet et al.^12^ demonstrated the possibility of monitoring near-surface acoustic sources from ships in the Mediterranean sea, Taweesintananon et al.^13^ and Matsumoto et al.^14^ compared DAS-recorded seismic airguns to traditional instrumentation, and Bouffaut et al.^15^ demonstrated the potential of DAS to monitor baleen whales. These studies revealed that the large number of sensors allows a high antenna gain from coherent processing, dramatically increasing the signal-to-noise ratio (SNR), while the long spatial extent allows near-field beamforming to focus on sources, delivering not only bearing but potentially range.

In recent work, Zhan et al.^10^ used the fiber optic sensing technology State Of Polarisation (SOP) to detect major earthquakes, measuring the integrated response along the entire 10,000 km length of the cable. However, SOP cannot currently localise sources or benefit from beamforming. DAS can also be used to detect acoustic signals, but, to the best of our knowledge, the farthest distance along a cable for which DAS has been reported is 171 km in a controlled experiment^11^. We used approximately the half length of an existing submarine FO cable that runs from Longyearbyen to Ny-Ålesund in Svalbard, Norway, shown as the blue line in Fig. 3. We connected the Longyearbyen end of the cable to an advanced DAS interrogator, recording data from about 120 km of a submarine FO cable at closely spaced intervals of a few metres and streaming data from Svalbard to NTNU in Trondheim in near-real-time. This represents both an innovation and a paradigm shift in how we can make distributed and coherent measurements moving the observatory itself to a virtual domain via web and cloud.

DAS offers the promise of not only sampling remote and inaccessible regions but also doing with unprecedented spatial resolution and extent at a moderate cost because the FO cables are already in place. The potential applications are countless. To demonstrate the versatility of DAS-recorded data, this article successively investigates DAS for ship, whale, earthquakes and storms monitoring—from the perspective of our Svalbard experiment.

Anthropogenic noise has significantly changed oceanic soundscapes over recent decades, adding to a long list of stressors that impact marine life^16^. The source characteristics of modern ships and their potential impact are still insufficiently characterised^17^. Following up on a study in Mediterranean Sea^12^, we investigate the potential of DAS to detect and track ships.

Despite the clear importance of cetaceans^18,19^, many species are still threatened or critically endangered, with insufficient data due to the difficulty of continuous monitoring^20^. The challenge is that many cetaceans cover large, partly inaccessible habitats with long migratory routes^21^, increasingly threatened by acoustic pollution, ship strikes, entanglement, contaminants and climate change^22^. These gaps exemplify our lack of adequate global detection and monitoring tools. We present the potential of DAS to reduce the data gaps for whale monitoring. The separately published full-length study, using data from the Svalbard experiment, demonstrated the capacities of DAS to record, detect and localise baleen whales, and showed the possibility to exploit whale non-stereotyped vocalisations to provide fully-passive conventional seismic records for subsurface exploration^15^.

Detecting small induced earthquakes is especially important for accurate monitoring of oil and gas production, wastewater injection, carbon dioxide (CO$_2$) sequestration, natural hazards and many other applications^23^. The high SNR, combined with coherent near-field beamforming, can reveal small seismic events that might otherwise go undetected. In addition, DAS can be a supplementary tool to constrain the localisation of earthquakes and help identify new features in the subsurface through ambient noise imaging.

Storms are significant ocean-atmosphere disruptions that could widely impact people and environment^24^. We investigate the potential of DAS to monitor distant storms that generate ocean swells. With data available in near-real-time from existing submarine telecommunication cables, DAS could be an attractive low-cost system for storm and tsunami monitoring to nicely complement existing sensing systems such as satellites (which are broadly limited to very near-surface observations), buoys, moorings, and floats (which have limited spatial coverage and resolution).

## Methods

**Figure 2:**
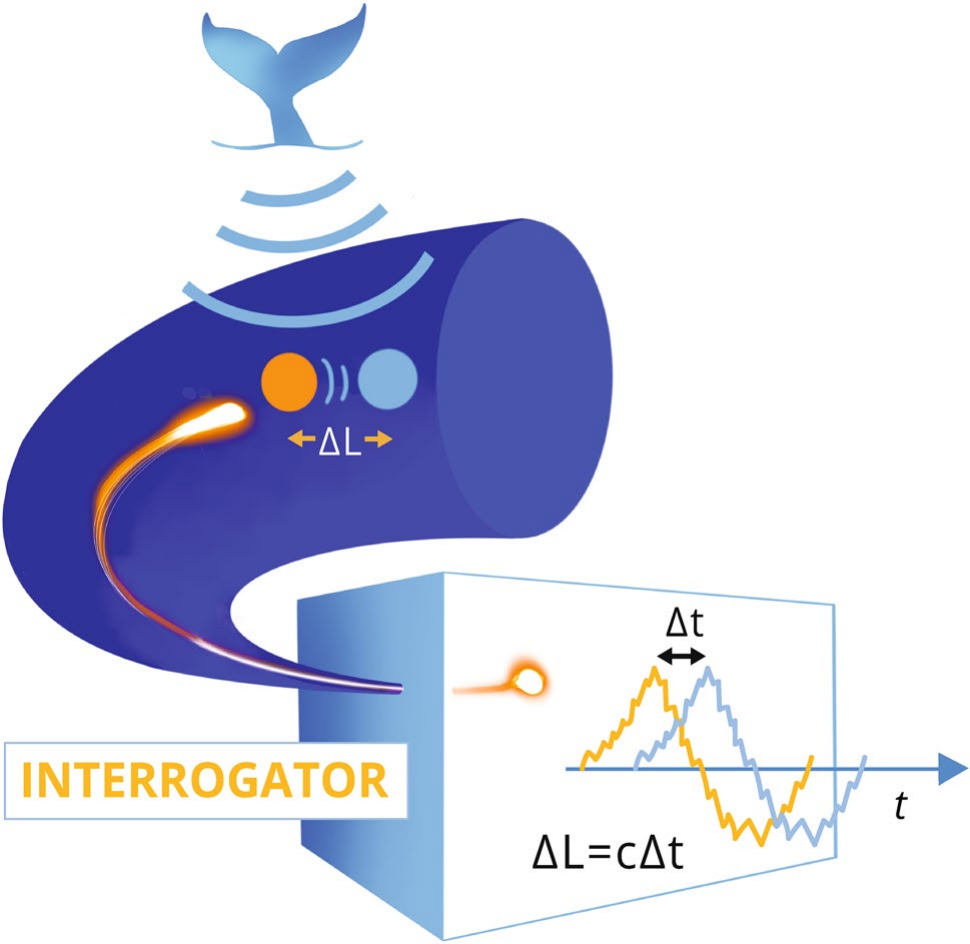
DAS measurement concept. The acoustic wavefield originating from the whale vocalisation modulates the fibre. The light propagating inside the fibre, which is backscattered due to inhomogeneities of the glass, is thus reflected from positions being shifted in space by the acoustic wavefield. This is indicated by the orange and light blue sphere. A DAS interrogator can reconstruct this acoustic wavefield present at each location along the fibre by detection of the change/modulation to the phase of the backscattered light, illustrated by the two shifted curves in the interrogator box, also colour-coded to see the relation to the two spheres that are shifted by the acoustic signal generated by the whale.

We used a dark (single-mode G.652D) fibre in an existing Uninett submarine telecommunication cable, installed into soft sediments at 0–2 m below the seafloor, running from Longyearbyen to Ny-Ålesund in Svalbard, Norway. The cable is double armoured containing 24 fibres (type G.652D) protected in a single, stainless steel tube. A polyethylene sheath is extruded around the single tube/stranded tube package to complete the cable core. A double layer of armouring wires is stranded around the cable core. This is in turn protected by a polyethylene outer sheath with cable’s outer diameter of 25 mm. We connected the Longyearbyen end of this cable to an Alcatel Submarine Networks ‘OptoDAS’ interrogator, streaming data from Svalbard to NTNU in near-real-time via the Uninett’s research network using the 1 Gbit/s network interface on the interrogator. The OptoDAS interrogator injected linear frequency-modulated optical pulses^11^ which are backscattered due to inhomogeneities of the glass in the fibre. The interrogator calculates the time-differentiated phase change of the backscatter response from consecutive sweeps for each sampled fibre position. The time-differentiated phase change can be converted to the longitudinal strain of the corresponding fibre section^25^. The DAS measurement concept is illustrated in Fig. 2. In this experiment, we used light pulses of 1550 nm free-space wavelength to sample 30,000 channels with 4.08 m spacing from 0 to 120 km along the cable. The gauge length used was 8.16 m, providing 8.16 m resolution along the cable. The signal strength decays along the cable, such that the returned signal strength from 100 km is $\sim -40$ dB with respect to 1 km. The DAS data were continuously recorded over 44 days and sampled with 645.16 Hz (1.55 ms interval), providing just over 320 Hz of bandwidth.

**Figure 3:**
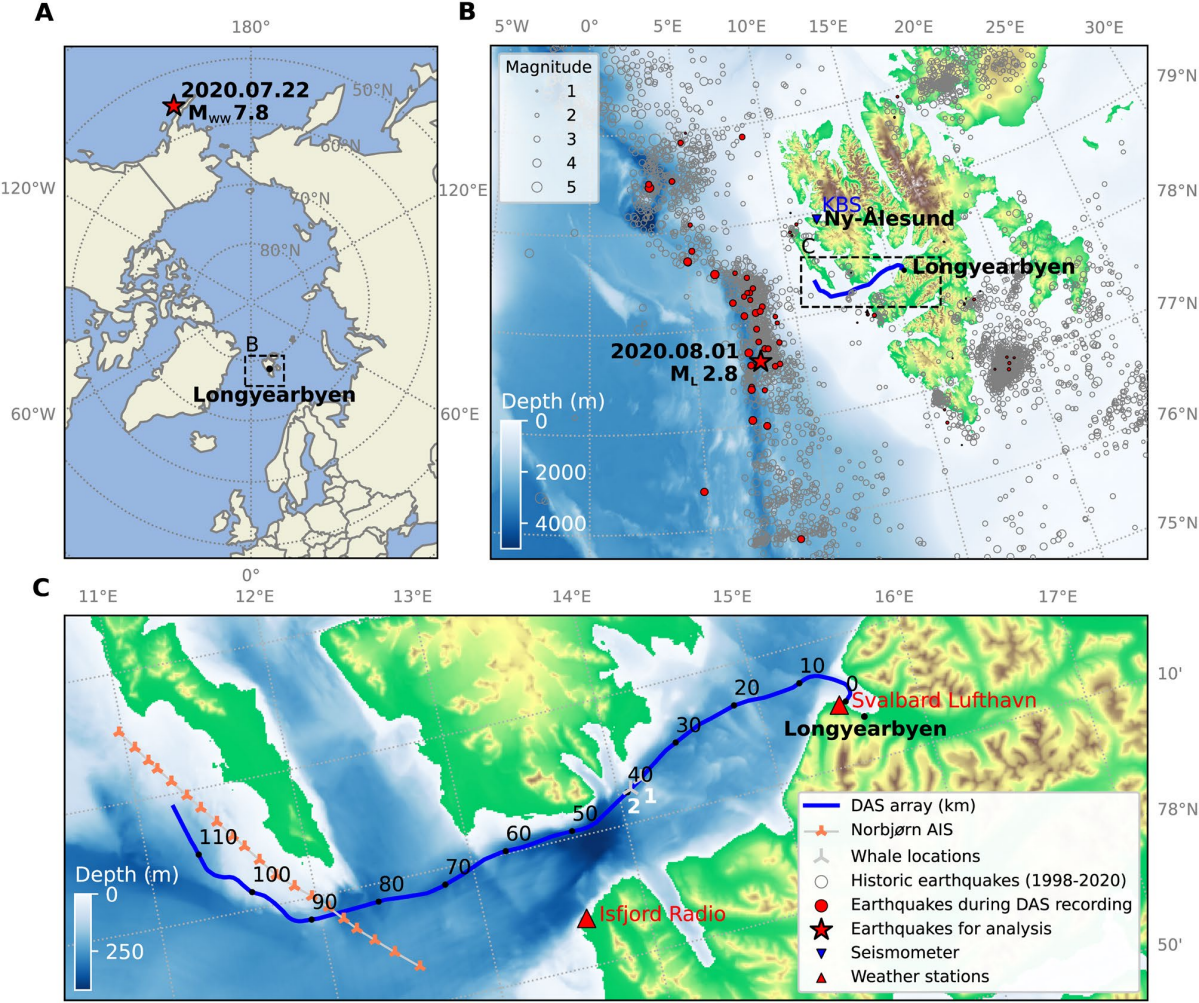
Geographical locations. (**A**) A polar stereographic map of the Arctic showing the epicentre of the $M_{ww}$ 7.8 teleseismic earthquake in Alaska on 2020.07.22 and the location of our FO array, near Longyearbyen. (**B**) A regional map illustrating the epicentres of the $M_L$ 2.8 earthquake in the mid-Atlantic ridge on 2020.08.01 and other recorded earthquakes between 1998 and 2020. (**C**) A local map showing 120 km of the underwater DAS array from the shore at Longyearbyen.

For ship tracking, vessel positions were estimated by optimising the cross-correlation of observed time-of-arrival on 2-second time windows with predicted time of arrival along the FO cable at 16 m spacing for an array of candidate positions. The usual left-right ambiguity associated with linear arrays was suppressed by choosing the solution that matches the Automatic Identification System (AIS) track, but could in principle have been resolved by the asymmetry in bathymetry or the vessel’s acoustic directivity.

For the earthquake analyses, we used several processing tools (band-pass filtering, *f*-*k* (frequency-wavenumber) filtering, *f*-*x* (frequency-space) swell noise attenuation, and *f*-*x* deconvolution) in addition to beamforming to increase the SNR of the recorded signal and better extract P- and S-wave phase arrival times. When beamforming, we first estimated the apparent velocity and angle. Then we used these values to find the optimal number of traces that could be stacked coherently for different segments of the cable. In this way, we were able to increase the SNR for a segment of the FO cable (spanning over 61 km along the FO cable) from 10.7 dB to 30.5 dB for the P-wave signal and 20.4 dB to 40.6 dB for the S-wave signal (Fig. S2).

For the distant storm analysis, we used the method given by^26^ based on^27^, whereby the time-frequency gradient can be used to calculate great-circle distances and travel times of the storm-induced ocean surface gravity waves from the storm centre to the DAS array.

## Results

### Ship and whale detection and tracking

**Figure 4:**
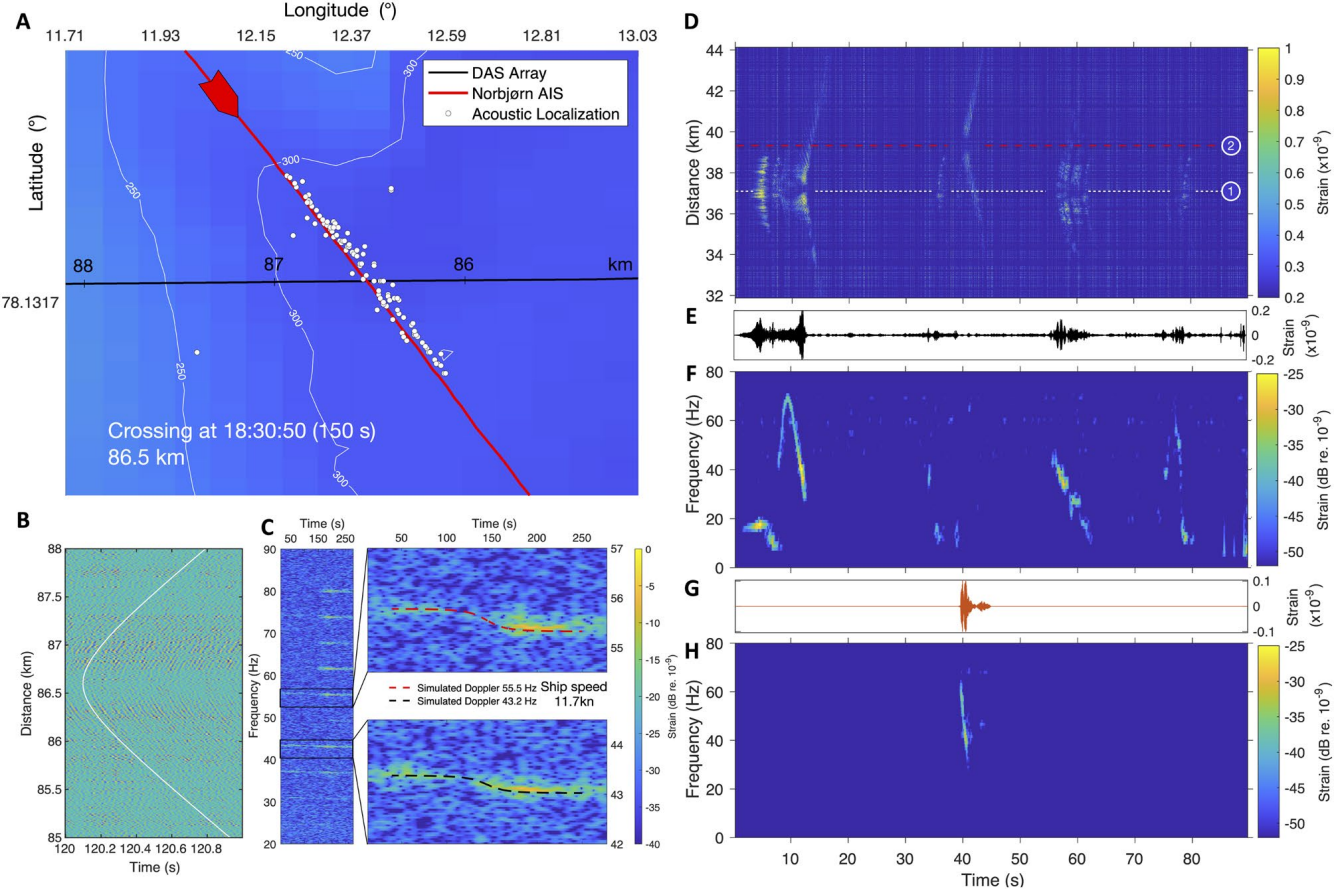
DAS detection, localisation, tracking and identification of a ship (**A**–**C**) and two whales (**D**–**H**). (**A**–**C**) General cargo ship Norbjørn was tracked as it headed SE at 11.7 kn in 300 m of water around 86.5 km of FO cable. (**A**) Comparison between acoustic localisation and Automatic Identification System (AIS) track. Norbjørn’s acoustic signature (**B**) in the space-time domain at the time of crossing, shows hyperbolic wavefronts used for acoustic localization (**C**) spectrogram, at 160 m from the crossing point, varies in time due to the Doppler effect. (D–F) Baleen whale calls over 12 km of FO cable (Audio S1). (**D**) Space-time representation used for identification and acoustic source separation: Whale 1 was at 37.2 km and Whale 2 was at 39.4 km. Waveforms (**E**,**G**) and associated spectrograms (**F**,**H**) after beamforming and source separation: Whale 1 (**E**,**F**) show a vocal repertoire characteristic of North Atlantic blue whales^28^ plus some likely-social D-calls (30–70 s) while the signal from Whale 2 (**G**,**H**) could be attributed to a fin whale.

During the sampling period, several ships passed close by and/or over the FO cable. One example is shown in Fig. 4A–C where we detected and tracked the Norbjørn, a general cargo ship (86.6 m overall length; dead weight tonnage 3389 T) proceeding SE at 11.7 kn. AIS position data reported by the ship are shown as a red line in Fig. 4A. Vessel positions estimated from the DAS data (white circles) correspond well with the AIS track, with a root mean square deviation of only $\pm 50$ m. Positions are estimated from the hyperbolic wavefronts arising from the curved nearfield propagation to the FO cable visible on Fig. 4B, allowing near-field beamforming to determine both range and bearing (with a left-right ambiguity). We also detected the Doppler shift as the vessel crosses over the fibre, from which we estimated the vessel speed of 11.7 kn, in close agreement with the AIS data. The Doppler spreading is clearly seen modulating the ship’s tonals in Fig. 4C.

Using data from the same experiment, Bouffaut et al.^15^ demonstrated the capacities of DAS for acoustic monitoring of baleen whales. Whale detections were made out to at least 95 km along the cable, and the analysis identified call types from at least two low-frequency baleen whale species: North Atlantic blue whale (*balaenoptera musculus*) and fin whale (*balaenoptera physalus*), with a number of stereotyped North Atlantic blue whale signals (AB call, peak frequency at $\sim 16.9$ Hz; arched sounds, 9-Hz call^28^) and mid-frequency down-sweeps (peak frequency $\sim 45 \pm 15$ Hz, average duration $5.4 \pm 2.4$ s). Down-sweep calls were the most common and can be attributed to blue whales (AB and D-calls), fin whales (D-calls or pulses), but also sei whales (*balaenoptera borealis*) or humpback whales^29^. Blue whale stereotyped calls were recorded during the entire recording period with a higher number of calls detected after 2020.07.23. In four instances, sightings from whale-watching tours confirmed the presence of a blue whale in the area. The sightings were reported on 2020.06.29 (1 individual), 2020.07.01 (1 individual), 2020.07.07 (3 individuals), and 2020.07.26 (1 individual). We identified downsweep vocalisations in the DAS data on the first two dates, and blue whale stereotyped calls in the DAS recordings on the two latter dates. A more detailed description of the sightings in relation to our recordings, including the sighting location and the time difference between DAS recording and sighting can be found in the supplementary materials (S1).

Figure 4D shows an example from within Isfjorden on 2020.06.27 at 17:35 UTC in which hyperbolic wavefront arrivals, whose apex indicate the point on the FO cable closest to the source, can be seen from two distinct whales, at 37.2 and 39.4 km. Figure 4F,H show spectrograms of the signals from the two whales with their respective waveforms obtained from beamforming (E &G). A stereotyped Atlantic blue whale call can be seen in Fig. 4E,F before 10 s, and arched sounds around 10 s and 78 s. Figure 4G,H show a short downsweep, likely from a fin whale. The high spatial resolution and extent of FO cables offer a complementary sensing modality to other cetacean monitoring techniques, including the ability to determine both range and bearing to animals, separating individuals aurally and spatially (Audio S1) and with the potential to track the movement of vocalising whales over continuous time periods over long distances.

### Earthquakes

We analysed seismic signals from a local and a teleseismic earthquakes at distances of $\sim$ 100 and $\sim$ 5,100 km, respectively, from the FO cable. These are depicted in Fig. 3 together with the region’s seismicity. We observe different wave types (compressional and shear) excited in the ocean sub-bottom that correspond to these seismic events as described in Saito & Tsushima^30^. As in previous studies^3–6^, our DAS analysis shows a rich expression of natural features, from 0.01–20 Hz, capturing not only the range and direction of earthquake hypocentres, but also the effect of deeper-diving waves passing through regions of higher velocity, and the interaction with local bathymetry^31^. We demonstrate that source localisation, exploiting the long array with a massive number of sensing points, represents a valuable complement to conventional methods. These results can be found in supplementary materials (Figs. S1–S3).

### Storms and ocean swell

**Figure 5:**
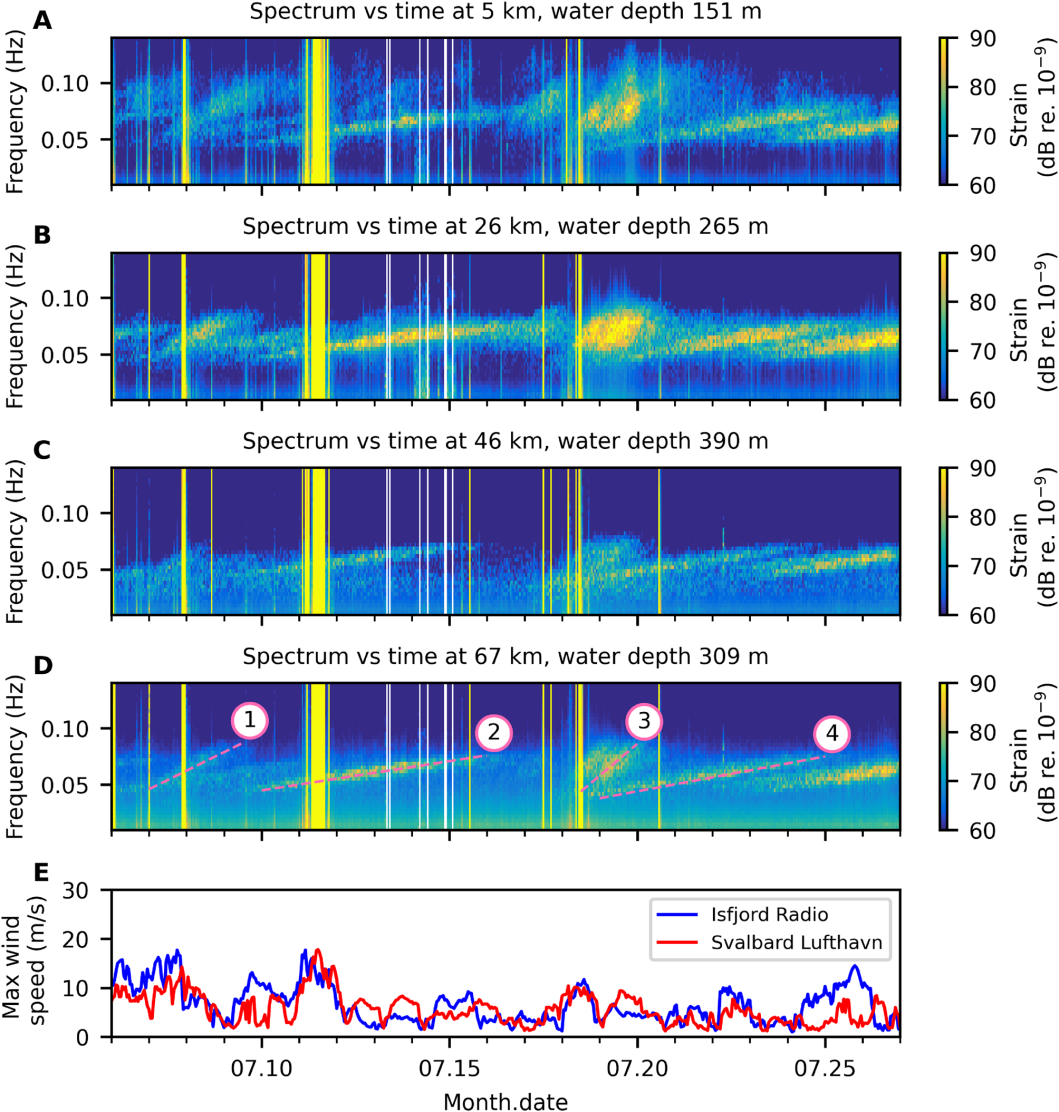
Atlantic Ocean storms observed from DAS. Spectrogram at 5, 26, 46 and 67 km of FO cable (**A**–**D**). Yellow vertical stripes are due to saturation, while the white vertical stripes were caused by data drop-out in the communication with NTNU. The bottom panel (**E**) shows the associated maximum wind speed measured at Isfjord Radio and Svalbard Lufthavn weather stations (Fig. 3). Four storm events marked in (**D**) are discussed in texts.

Figure 5A–D display spectrograms of 21 days of data at four different points along the FO cable, showing nearly-linear narrow-band dispersion trends with different gradients, ranging from 0.04–0.10 Hz over several days. These trend lines are generated by ocean surface gravity waves from distant storms. The trend lines were also observed in the data recorded by a conventional ocean bottom sensor^32^. Figure 5E shows wind measurements at two weather stations: Isfjord Radio, which is at $\sim 55$ km from the entrance of the fjord and Svalbard airport, where the cable begins.

We observe that the trend lines increase monotonically and linearly in frequency over time. During the entire 44-day recording period, we can easily identify 12 such trends in the time-frequency spectra. Each of these events typically lasts for 50–100 hours, with some overlapping. The amplitude level of these events increases towards the inner parts of the fjord, which implies that the fjord acts as a narrowing amplifier for this type of signal.

Four linear trends corresponding to distant storms are highlighted in Fig. 5D. We used the time-frequency gradient to calculate great-circle distances and travel times of the storm-induced ocean surface gravity waves from the storm centre to the DAS array, allowing us to trace all four events back to the generating storms. Event 1 corresponds to the Tropical Storm Edouard $\sim$ 4100 km from Longyearbyen, occurring on 2020.07.04–06^33^. Event 2 possibly corresponds to the bomb cyclone in offshore south Brazil at about 13,000 km from Longyearbyen from 2020.06.30 to 2020.07.02 as reported in^34^ and^35^. According to weather news in Iceland^36^, Event 3 should correspond to an extratropical depression between Iceland and Greenland at about 2400 km away from the DAS array from 2020.07.15–17. Lastly, Event 4 probably comes from a storm in a remote region in offshore south Brazil at about 11,000 km from the DAS array on 2020.07.12. These four events are examples from the total 12 events observed in our data set. Many events like Event 4 and the stronger trend in the rightmost of the time-frequency spectra in Fig. 5D come from remote regions in the South Atlantic Ocean between the eastern coast of South America and the western coast of Africa. The storms in these remote regions might not affect human and, hence, not be documented. However, they are obviously detected by DAS. Thus, DAS could be a potential storm monitoring system with global coverage.

## Conclusion and discussion

The wide range of valuable monitoring capabilities that DAS now offers, especially if extended to near-continuous monitoring over many FO cables, spanning the globe, is a game-changer. To realise the potential of a cloud-based global observatory illustrated in Fig. 1 will require data management and automated real-time analysis on an unprecedented scale. The 120 km length of FO cable sampled for this work generated $\sim 7$ TB of data a day.

The ability to sample the acoustic field over a wide frequency range (four orders of magnitude) coupled with the high spatial resolution (a few metres) and extended aperture (over 100 km), provides unprecedented discriminatory power. The extended aperture allows near-field beamforming, yielding not only a bearing but also range of point-like sources near the cable. The high spatial resolution allows higher frequencies to be analysed without aliasing. The broad frequency band permits us to uniquely characterise different source types, distinguishing distant storms, earthquakes, ships and whales, which may be received simultaneously. Distant storms leave an acoustic ‘fingerprint’ in the 0.01–0.1 Hz frequency range. Earthquakes produce pressure and shear waves in the 0.01–10 Hz range, and local events can be localised in range and azimuth direction. Ships produce nearly-continuous signals in the 35–85 Hz range and can also be localised, while baleen whales produce repeated stereotypical time-frequency transients that are easily differentiated from ships and which can also be simultaneously located and separated in range and bearing.

The ship example shows that vessels passing over or near a FO cable can be detected and tracked, using near-field beamforming, and that there is even a potential to characterise the acoustic signature and directionality, including identification, if signals are routinely acquired from an array of FO cables, augmented by AIS data.

The baleen whale example further shows that the high SNR gain available from extended aperture array processing can be used to construct high-quality audio waveforms. We expect to be able to detect whale vocalisations for distances along a FO cable in excess of 100 km. DAS, combined with AIS and other data streams, could generate real-time whale- and ship-tracking with early warning and alert systems broadcasted over the existing AIS system to vessels that could then reduce the risk of ship-strike injuries, recognised as one of the most critical anthropogenic threats to cetaceans^22^ and anthropogenic noise impacts, a considerable, yet difficult-to-manage threat to cetacean populations^37^.

The earthquake studies demonstrate the possibility to use FO cables to estimate the ratio of compressional to shear wave velocities, $v_p/v_s$, from different segments along the cable as well as to localise a local earthquake to the position matched with the conventional networks. In addition, DAS reveals ultra-low frequency ($\sim 0.01$ Hz) pressure loading response to ocean surface gravity waves from local winds and distant storms.

In conclusion, we believe that DAS has now reached a performance level that offers a disruptive capability in global Earth-Ocean-Atmospheric-Space monitoring, providing a hitherto unimaginable spatial coverage and resolution, combined with continuous data streaming. The broader implications include:

- Long-term climate monitoring: DAS could provide spatial and temporal dimensions to complement other measurement schemes, ‘averaging out’ point sampling noise in space and time to give more accurate measurements of small changes, as they were achieved with satellite altimetry and Global Positioning System (GPS) by using many passes to get millimetre accuracy of position, sea and terrain heights. It could also be possible to monitor ice processes such as fracturing and calving in the Arctic.
- Long-term monitoring of CO$_2$ sequestration reservoirs: CO$_2$ sequestration is an important element in the energy transition and move to decarbonise. DAS has already been applied for CO$_2$ storage monitoring to map CO$_2$ plumes at two pilot projects^38^. A combination of downhole and seabed horizontal fibres has a great potential for reducing monitoring costs compared to conventional time-lapse seismic.
- Atmosphere-Ocean interactions: A topic that is still not fully understood, where DAS offers something new in its ability to continuously monitor ocean swells from distant storms with global coverage.
- Monitoring the ocean soundscape: DAS can sample in places where there has been little interest or resource support, e.g. off the coasts of South America and Africa, mitigating geospatial and societal biases in observations in line with UN sustainable development goal (SDG) 9.5, enhancing scientific research and upgrading technological capabilities and applications, particularly in developing countries, and SDG 14.a, improvement of ocean health and enhance biodiversity in developing countries.
- Big Data: DAS offers the potential of collecting a truly Big Data set, with extensive spatial and temporal coverage, ripe for exploitation by data mining and artificial intelligence (AI) to reveal global patterns. Because DAS simultaneously measures processes occurring in the atmosphere, ocean and solid earth below, there is the potential to discover cross-disciplinary interactions and global patterns between meteorologists, oceanographers and geophysicists.

The biggest challenges lie in improving the SNR and range capabilities, the availability of dark fibres (or the ability to use ‘live’ fibres), reducing the cost of interrogators, data management, and applying automated detection and classification methods. None of these challenges are without solutions and work is rapidly pushing back the boundaries of what is possible, which will lead to a near-future breakthrough in continuous, distributed environmental sensing over the majority of the planet’s surface.

In 2022, we plan a new DAS survey at Svalbard, using two FO cables that are separated by typically some hundred metres along the track shown in Fig. 3. This enables a richer possibility to track whales and ships and waves generated by distant storms. Four interrogators will be used to obtain DAS data covering a total distance of approximately 250 km. In this experiment we will also use polarimeter measurements to complement the DAS measurements.

## Supplementary Information

Supplementary Information 1.

Supplementary Information 2.

Supplementary Information 3.

## Data Availability

AIS data obtained from Kystdatahuset data provider https://kystdatahuset.no. Whale sighting data provided by Svalbard adventures Turproduksjon https://svalbardadventures.com and Henningsen Transport & Guiding https://www.svalbardcruise.com. Seismic data from the station at Svalbard are available on the IRIS web Services: https://service.iris.edu/. Details on the Alaska earthquake available at: https://earthquake.usgs.gov/earthquakes/eventpage/us7000asvb/executive. Historic seismic data near Svalbard available at: https://www.norsar.no/seismic-bulletins/ and http://nnsn.geo.uib.no/nnsn/#/data/events/bulletins.
