# Supplementary material for: Sensing whales, storms, ships and earthquakes using an Arctic fibre optic cable
Source: Sci Rep. 2022 Nov 10;12:19226. doi: 10.1038/s41598-022-23606-x (PMC9649797; doi:10.1038/s41598-022-23606-x)
Supplement: Supplementary file 3 — Supplementary Information 3. [file 41598_2022_23606_MOESM3_ESM.pdf]

Supplementary Alaska and local earthquake.

On 2020.07.22 at 06:12:44 UTC an M_{ww} 7.8 earthquake occurred approximately 100 km south of the Alaska Peninsula at a depth of 28 km. The earthquake was recorded at seismic stations worldwide including our DAS array near Longyearbyen, ~5100 km away from the epicentre on a great circle. Figure 2(**A**) shows the location of this earthquake, while the inset (**B**) shows the DAS array and the KBS seismic station, belonging to the Global Seismograph Network (GSN), at Ny-Ålesund. The DAS data have similar characteristics to the east-west vibrating component recorded by the KBS seismometer. Figure S1(**A**) compares the seismogram from a single DAS channel (top) at 36 km distance from the shore with a stacked version (middle) from the sum of 25 neighbouring traces, and the seismogram from the KBS east-west component (bottom). There is an excellent correspondence of P, S and SS arrivals between the data from DAS and the KBS component. Stacking 25 traces increases the SNR of the DAS data by $\sim$ 3 dB, still below the 34 dB SNR offered by the KBS seismometer. The advantage of DAS over a conventional seismometer lies in the length of the array of sensors, in this case 120 km. With such an extended array, we can visualise teleseisms in a space-time display, where coherent teleseismic events are easily observed and distinguished. Here, we emphasise that by analysing the 2D profile we can easily identify the surface waves corresponding to the Alaska earthquake and a separate P wave arrival from another source, hardly distinguished in the KBS east-west seismogram.

A local M_L 2.8 earthquake with epicentre close to the Mid-Atlantic ridge occurred on 2020.08.01 at 02:54:00 UTC. Figure S1(**B**) shows the signal in the space-time domain. The earthquake seismic waves arrive at the FO cable from a nearly-orthogonal direction compared to

the Alaska teleseism, which arrives from a broadside direction. This offers a unique possibility to compare array effects and analyse apparent velocities for P and S waves from the two earthquakes. For the local earthquake, we find that the apparent velocity and the ratio of P- to S-wave velocities (v_p/v_s) increases with the distances from the epicentre to the DAS sensors. This is due to energy propagation eigenpaths travelling at increasing depth to reach sensor points at increasing distance from the epicentre.

Because the Atlantic earthquake is closer to the cable (110–190 km away) compared to the Alaska earthquake (5100 km away), we can use the relatively long array (120 km) to estimate the position of the Atlantic earthquake, which is then compared with the position reported by the Norwegian National Seismic Network (NNSN) and NORSAR. We applied an optimisation algorithm to estimate the epicentre, which resulted in a location in close agreement (~10 km) with that reported by NNSN and NORSAR. Previously, Jousset et al. (*20*) relocated a local earthquake using DAS, while Nishimura et al. (*28*) located volcanic earthquakes using DAS and grid search methods. Nevertheless, to the best of our knowledge, this is the first example of using DAS data from a 100+ km-long FO cable to localise an earthquake (Figure S3). The long antenna offered by FO cables, especially if they are curved, offers a unique complementary method to traditional source localisation for earthquakes and other seismic sources.

We also analysed the data down to a frequency of 0.01 Hz, which reveals additional environmental insights. Figure (Figure S4) shows three power spectral density levels from 0.01–20 Hz, computed over three different 300 s time windows. The DAS data in Figure S4(**A**) contain no known significant earthquakes. Therefore, this time window represents background microseismic noise. Figure S4(**B**) contains the primary P-wave arrivals from the M_{ww} 7.8

teleseismic earthquake from Alaska on 2020.07.22. The spectrum in Figure S4(**C**) is computed from a time window that contains the primary P-wave and S-wave arrivals from the M_L 2.8 earthquake from the mid-Atlantic ridge on 2020.08.01.

In the background microseismic noise data in Figure S4(**A**), we clearly see two types of signals. The first is the so-called primary microseism, with frequencies from ~0.04–0.10 Hz, whose spectral density is independent of water depth. This signal is generated by variations in the loading pressure associated with sea surface height. It is, therefore, a hydrostatic response associated with ocean surface gravity waves excited by winds or a nonlinear wave-wave interaction mechanism (*28*). The phase velocity (c_p) of an ocean surface gravity wave is formulated as $c_p = \omega/k$, where the well-known dispersive relation (*30,31*) is $\omega^2 = gk \tanh(kH)$, ω is the angular frequency, k is the angular wavenumber, and H is the water depth. The second signal type has higher frequencies, ranging from ~1–10 Hz, and it is strongly correlated with the water depth. We interpret this signal to be an acoustic resonance of the water column (*32*). This dispersive hydroacoustic wave has cutoff frequencies for the rigid ocean bottom given by $f_n = (2n - 1)c/(4H)$, where n is an integer mode order and c is sound speed in the water column (*33*). The primary microseism and the fundamental mode ($n = 1$) of the acoustic resonance can be observed in Figure S4(**A**). The excitation mechanisms of the ocean bottom corresponding to different wave types are described in (*19*). The primary microseism (~0.04–0.10 Hz) and the hydrodynamic response (~0.1–1.0 Hz) show no correlation to the water depth, while the normal modes (~1–10 Hz) show a strong correlation to the water depth.

The number of excited modes typically increases for forcing signals with higher horizontal wavenumbers and with stronger acoustic sources. In Figure S4(**A**), we interpret the first high-

amplitude event as the first normal mode, and the source is most probably non-linear wave-wave interaction of long ocean waves, or minor earthquakes or other signals coming from the earth, or a combination of these. With the additional significant seismic energy in Figures S4(**B, C**), the hydroacoustic waves are stronger, and the higher modes of the acoustic resonance are more visible than those revealed by the background microseismic noise alone. In addition, the sea bottom pressure variation is accentuated when the seismic P-waves arrive. This hydrodynamic response can be observed between ~0.1–1.0 Hz, as clearly shown in Figure S4(**B**). Additionally, in Figure S4(**B**), we see that the Alaska earthquake leads to a significant amplitude increase, but with a similar number of excited modes as for the background microseismic noise. However, the mid-Atlantic earthquake in Figure S4(**C**) shows a larger number of excited normal modes, which attribute to the wave containing higher horizontal wavenumbers arising because the source direction was nearly in-line with the FO cable. The levels are also lower than those in the Alaska signal in Figure S4(**B**). Our DAS analysis therefore shows a rich expression of natural features, from 0.01−20 Hz, capturing not only the range and direction of earthquake hypocentres, but the effect of deeper-diving waves passing through regions of higher velocity, and the interaction with local bathymetry.

For the distant storm analysis, we used the method given by (*34*) based on (*35*), whereby the time-frequency gradient can be used to calculate great-circle distances and travel times of the storm-induced ocean surface gravity waves from the storm centre to the DAS array.

Extended data:

Figs. S1 to S5

Audio S1 to S2

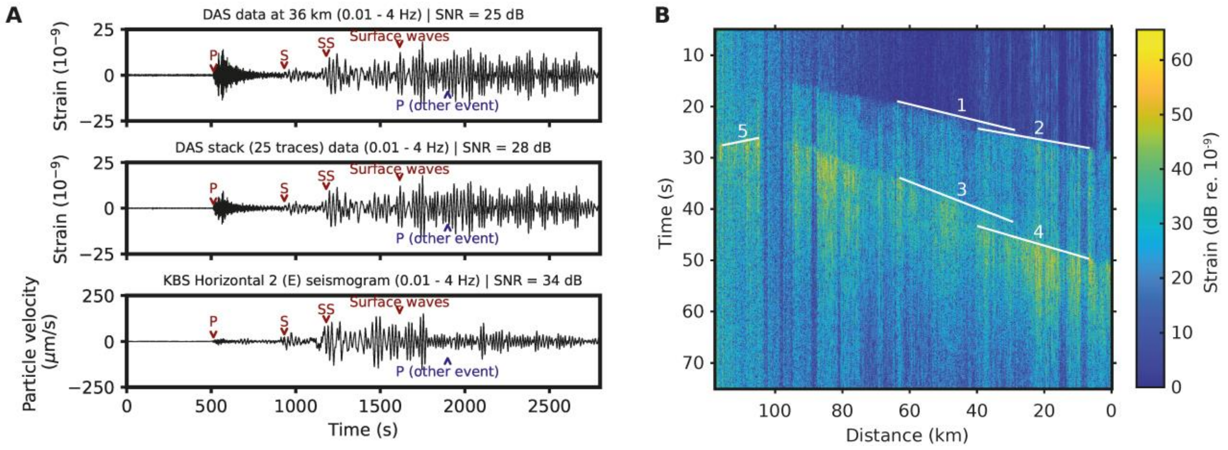

Fig. S1.

Seismic signals from a teleseismic Alaska earthquake (A) and a local mid-Atlantic earthquake (B). (A) shows a single DAS channel at 36 km (top), a stacked waveform using 25 traces around the 36 km channel (middle) and the east-west component from the KBS seismic station (bottom). (B) shows the local mid-Atlantic ridge earthquake signal over the entire 120 km long cable (plotted as time after origin time; **Audio S2**). The P- and S-waves are clearly visible. At approximately 22 km distance we observe a 15–20 km "pinch out" for both the P and S waves and different apparent velocities for different parts of the cable (white lines). Correcting for the apparent angle (~172.3˚) the apparent velocity of the pinch-out is 8.66 km/s (2) and 5.14 km/s (4), while the other observed apparent velocities are 6.21 km/s (1) and 3.76 km/s (3) with a correction angle of ~175.3˚. These values correspond to v_p/v_s ratios of 1.69 and 1.65, respectively. A third apparent S-wave velocity can be observed at the far end of the cable (5) with an apparent velocity of 2.23 km/s (correction angle of ~287.3˚).

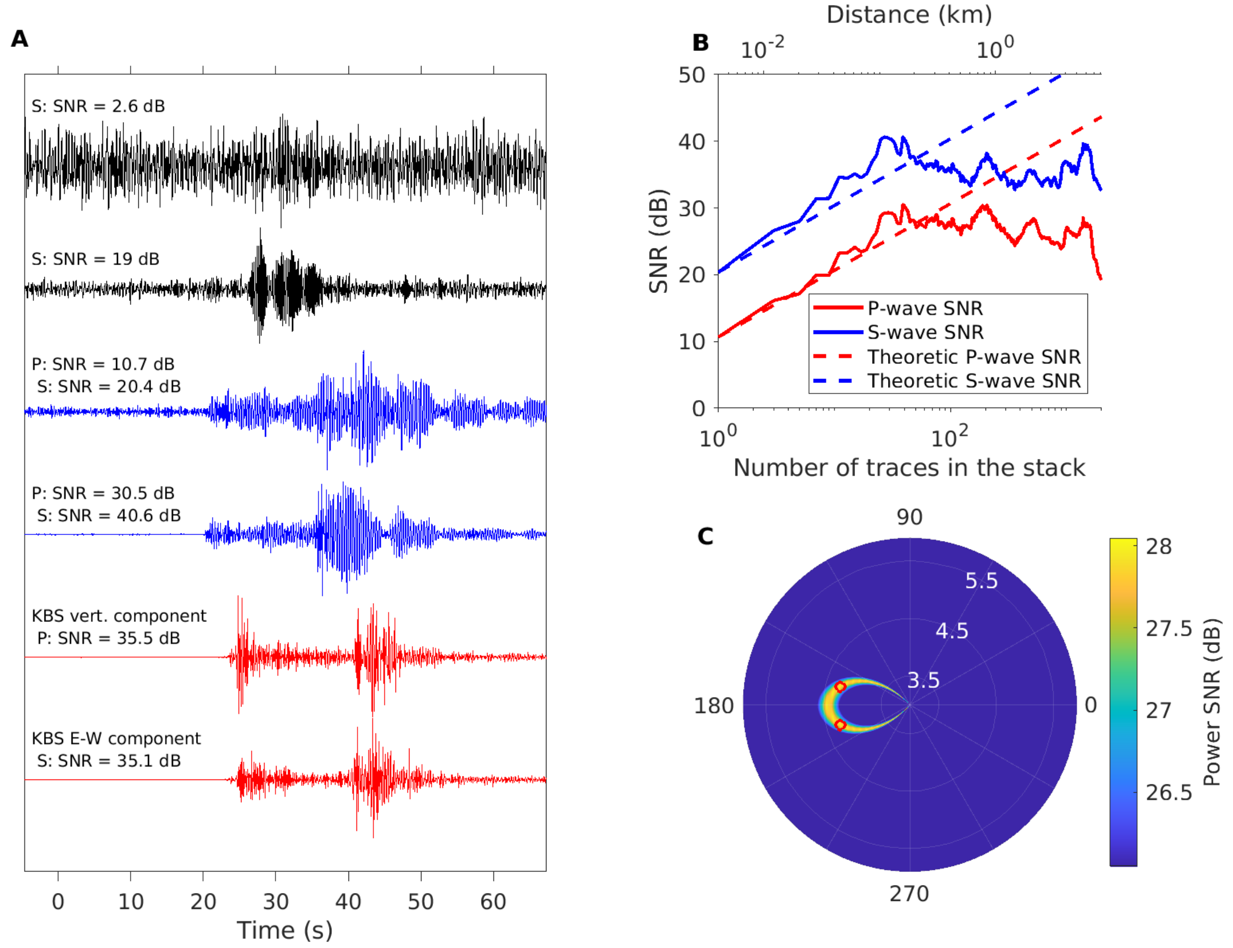

Fig. S2.

Increasing the SNR by finding the optimal apparent velocity and angle, and optimal number of traces in the stack that maintains signal coherence. (**A**) Comparison between single trace and a stack of 39 traces for channels at 114 km (black, high background noise level) and at 61 km (blue, low background noise level), against the vertical and west-east component of the KBS seismometer data (red). All traces are normalised relative to their absolute maximum. Stacking allows us to significantly improve the SNR and extract seismic signals otherwise buried in noise. The time-shift between the DAS and the KBS signal is caused by the difference in epicentral

distance (Figure 2). (**B**) SNR versus stacking length (in number of traces) for P- and S-waves around 61.3 km. Exceeding 39 traces reduces SNR. Dashed line corresponds to a $\sqrt{N}$ model for the improved SNR. (**C**) Beamforming for apparent P- and S-wave velocity and incidence angle using channels between 58.0-64.5 km with the same middle channels as the analysis in (**B**). The highest SNR values are found for angles of 193° and 192° relative to the cable axis, with velocities 4.25 and 2.45 km/s for P- and S-wave, respectively.

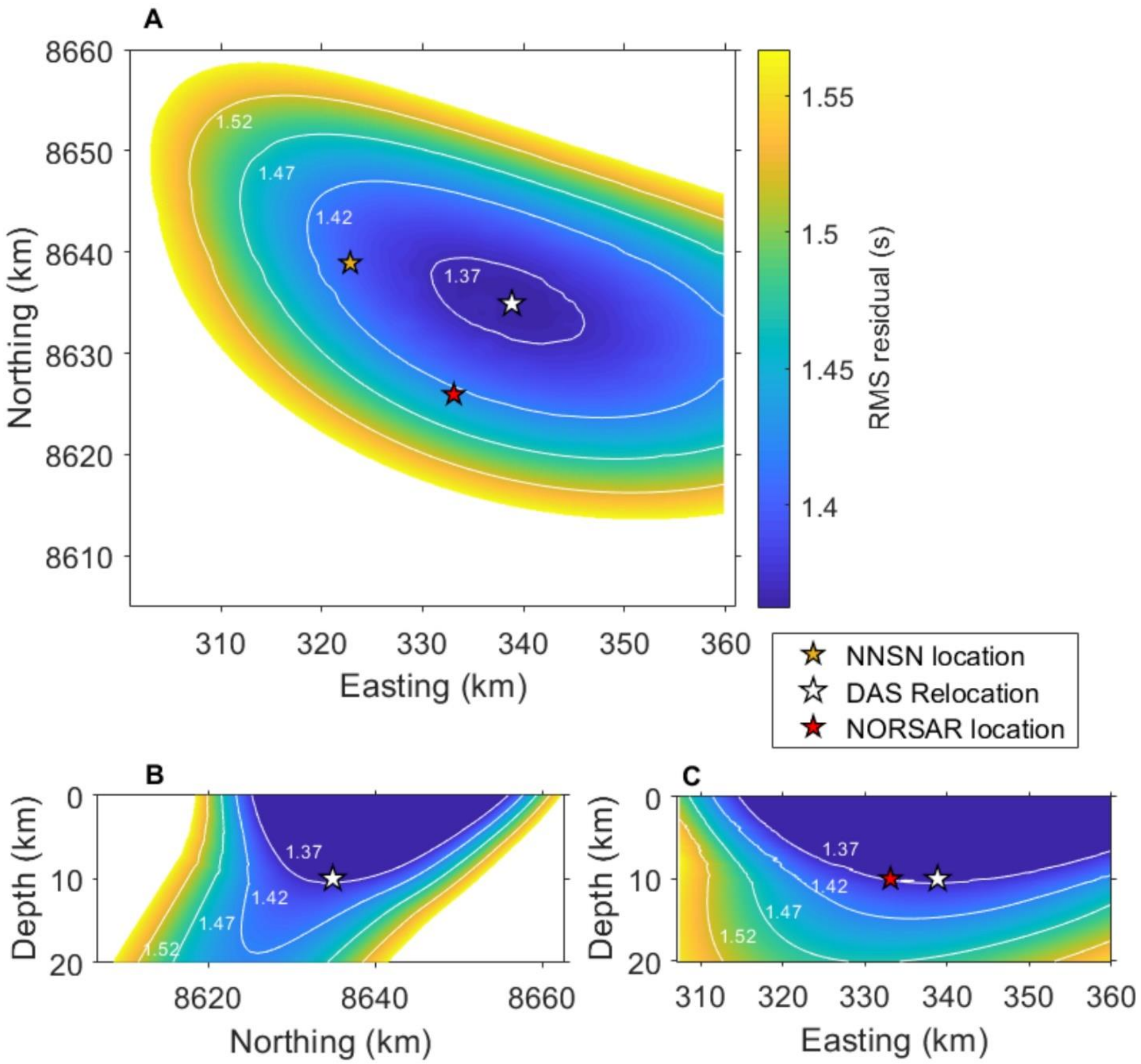

Fig. S3.

Localisation of the M_L 2.8 local earthquake using 53 DAS sensor points. The earthquake is relocated (white star) approximately 16.49 km south-east of the catalog location reported by NNSN (yellow star) and 10.65 km north-east of the NORSAR reported location (red star). The RMS of the relocated earthquake is 2.6% lower than the NNSN catalog location using ray tracing

to compute the eigenrays and 4.7% lower than the NORSAR catalog location. We used the dominant v_p/v_s = 1.65 ratio observed from the cable to estimate the S-wave travel time. (**A**) Shows the RMS values in the x-y plane for 10.0 km depth. (**B**) Shows the y-z plane through the DAS relocation. We observe a large uncertainty in depth towards the surface. (**C**) Shows the x-z plane through the DAS relocation, also showing the high depth uncertainty.

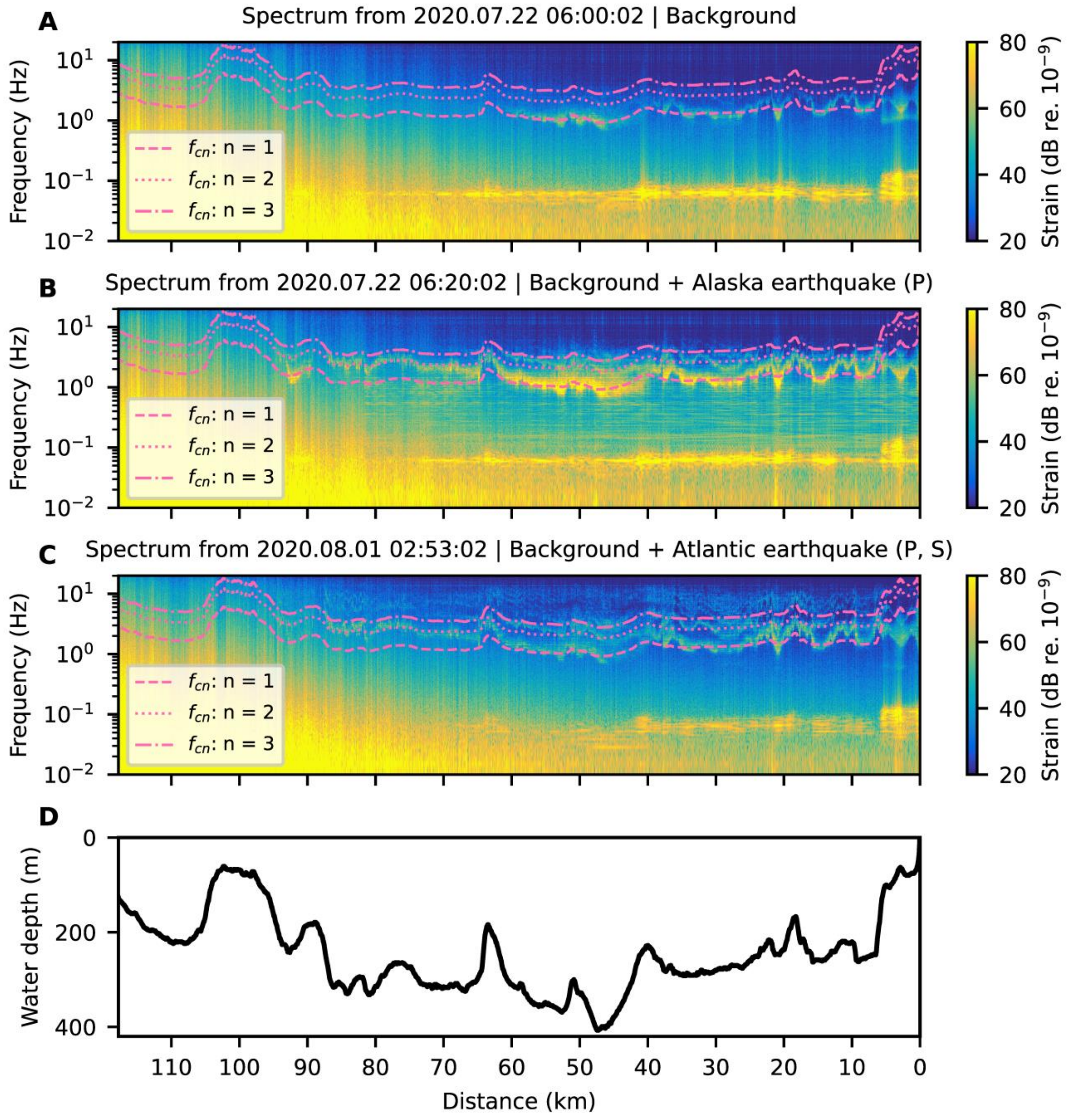

Fig. S4. Bathymetry and modal cut-off revealed by DAS.

Power spectral densities versus distance for (**A**) background microseismic noise, (**B**) during the arrival of the teleseismic P-wave signals from Alaska earthquake, and (**C**) during the Atlantic margin earthquake. (**D**) shows the water depth profile, which is correlated with the signals around

1–20 Hz. The plots are overlaid with the frequency functions defined by $f_{cn} = nc/(4H)$, where n is an integer, c = 1490 m/s is sound speed in water, and H is the water depth.

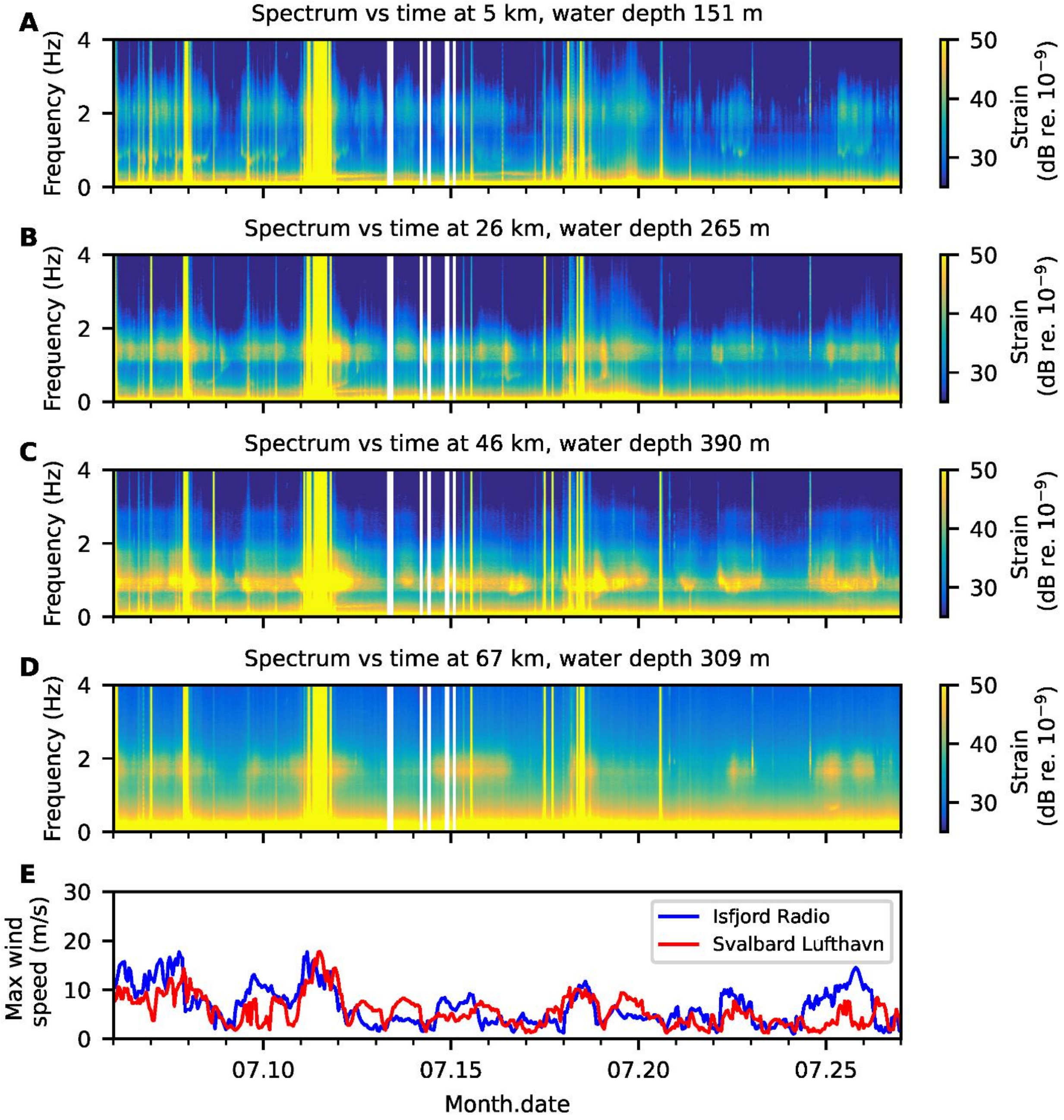

Fig. S5. Signal intensity correlated with local wind conditions.

Spectrograms (**A-D**) at 5, 26, 46, and 67 km along the cable. The vertical yellow bands show energy saturation associated with high local wind conditions (**E**). White vertical lines are break down in the data-stream.

Audio S1.

North Atlantic blue whale and fin whale vocalisations recorded on DAS cable on 2020.06.27. Blue whale arched sounds at 10 s and 75 s, possible D-calls at 30–70 s; possible fin whale vocalization at 40 s. Frequency scaled up by a factor of 3.5.

Audio S2.

Earthquake sound on 2020.08.01. Frequency scaled up by a factor of 20, from 125 Hz to 2500 Hz.
